# The variant T allele of *PvuII* in *ESR1* gene is a prognostic marker in early breast cancer survival

**DOI:** 10.1038/s41598-021-82002-z

**Published:** 2021-02-05

**Authors:** Danny Houtsma, Stefanie de Groot, Renee Baak-Pablo, Elma Meershoek -Klein Kranenbarg, Caroline M. Seynaeve, Cornelis J. H. van de Velde, Stefan Böhringer, Judith R. Kroep, Henk -Jan Guchelaar, Hans Gelderblom

**Affiliations:** 1 Department of Medical Oncology, Leiden University Medical Center, Albinusdreef 2, P.O. Box 9600, Leiden, 2300 RC The Netherlands; 2 Department of Clinical Pharmacy and Toxicology, Leiden University Medical Center, Leiden, The Netherlands; 3 Department of Surgery, Leiden University Medical Center, Leiden, The Netherlands; 4 Department of Medical Oncology, Erasmus MC Cancer Institute, Rotterdam, The Netherlands; 5 Department of Biomedical Data Sciences, Leiden University Medical Center, Leiden, The Netherlands

**Keywords:** Cancer, Genetics

## Abstract

The *PvuII* (rs2234693) Single Nucleotide Polymorphism (SNP) in the gene coding for the estrogen receptor-1 (*ESR1*), has been found associated with outcome in tamoxifen treated patients with early hormone-receptor positive breast cancer. However, it remains unclear whether this SNP is a predictive marker for tamoxifen efficacy or a prognostic marker for breast cancer outcome. The aim of this study was to examine the prognostic potential of this SNP in postmenopausal early breast cancer patients treated with adjuvant exemestane. Dutch postmenopausal patients randomised to 5 years of adjuvant exemestane of whom tissue was available (N = 807) were selected from the Tamoxifen Exemestane Adjuvant Multinational (TEAM) trial database. The SNP rs2234693 in the *ESR1* gene was genotyped on DNA from formalin-fixed paraffin embedded (FFPE) tumor tissue using Taqman assays and related to the primary endpoint disease-free survival (DFS) and secondary endpoint overall survival (OS). Survival analyses were performed using Cox regression analysis. In total 805 patients were included in the analyses (median follow up of 5.22 years) and genotypes were obtained in 97% of the samples. The variant T allele of *PvuII* in *ESR1* (rs2234693) was associated with a better DFS (hazard ratio (HR) 0.689, 95% confidence interval (CI) 0.480–0.989, *P* = *0.044*) in univariate analysis only, and a better OS in both univariate (HR 0.616, 95%, CI 0.411–0.923, *P* = *0.019*) and multivariate analyses (HR 0.571, 95% CI 0.380–0.856, *P* = *0.007*), consistent with a prognostic rather than a predictive drug response effect. Variation of *PvuII* in the *ESR1* gene is related to OS in postmenopausal, early HR + breast cancer patients treated with exemestane in the TEAM study. Variation in the *ESR1* gene may therefore be a prognostic marker of early breast cancer survival, and warrants further research.

## Background

Endocrine treatment is the cornerstone of treatment of hormone receptor positive breast cancer^1^, and for many years, tamoxifen has been the gold standard adjuvant endocrine therapy^2^. Over the last decades, however, in postmenopausal women third generation aromatase inhibitors have consistently demonstrated superior efficacy over tamoxifen, and optimal treatment is sequential use of tamoxifen followed by an aromatase inhibitor or an aromatase inhibitor alone^1,3–5^. Unfortunately, still 40% of women using optimal endocrine therapy experience a relapse^6^. To date, only the presence of hormone receptors are predictive markers for outcome^7^. Identification of more accurate biomarkers remains crucial to predict which women are responsive to endocrine treatment and the optimal therapy^6,8^. In a pharmacogenetic analysis of the Tamoxifen Exemestane Adjuvant Multinational (TEAM) study an association between tamoxifen efficacy and the rs2234693 SNP in the estrogen receptor-1 (*ESR1*) gene has been found in postmenopausal hormone receptor positive breast cancer patients, whereas patients with an increasing number of C alleles of *PvuII* in *ESR1* had a decreased disease free survival (DFS)^9^. Moreover, in a case–control study patients carrying the CC or CT genotype had a 4.14-fold increased relative risk to develop breast cancer^10^. Results from other studies on breast cancer risk or survival after breast cancer are conflicting, which may be explained by differences in hormone receptor expression, menopausal status or ethnicity^11–14^. Therefore, it remains unclear if this biomarker is predictive or prognostic, i.e. the latter being independent of treatment.

In order to investigate the value of this biomarker further, the aim of this TEAM substudy was to examine the prognostic potential of this SNP in postmenopausal, early breast cancer patients treated with adjuvant exemestane alone.

## Methods

### Study population

For the current pharmacogenetic substudy, data and tumor tissue of Dutch patients treated with exemestane alone enrolled in the Tamoxifen Exemestane Adjuvant Multinational (TEAM) trial were used. From January 2001 until January 2006 women participated in the multinational TEAM trial, randomizing between 5 years adjuvant exemestane (25 mg once a day, orally) or 2.5–3 years tamoxifen (20 mg once a day, orally) followed by exemestane for a total of 5 years^6^. Eligible patients had histologically confirmed hormone receptor positive early breast cancer, postmenopausal status and completed local treatment with curative intent. Other inclusion and exclusion criteria have been described elsewhere^6^. Informed consent was obtained from all individual participants included in the study. The international TEAM study was conducted in accordance with the Declaration of Helsinki and was approved by the respective institutional Ethics Committees whereof the Dutch part was approved by the central medical ethics review board of the Erasmus University Medical Center in Rotterdam. The current pharmacogenetic study was separately approved by the central medical ethics review board of the Erasmus University Medical Center in Rotterdam, The Netherlands in accordance with the Declaration of Helsinki.

### Endpoints

The primary endpoint of the current substudy was DFS, defined as time from date of randomization until the date of the earliest documented locoregional or distant recurrence, ipsilateral or contralateral breast cancer excluding ductal carcinoma in situ, or death from any cause. A secondary endpoint was overall survival (OS), defined as time from randomization until date of death from any cause.

### Genotyping

For genotyping tumor-negative tissue was used, however, when this was unavailable tissue from tumor-containing blocks were used. DNA was extracted from the FFPE tumor tissue as described earlier^15^. Briefly, three slides of 20 μm were used to extract DNA from with the Maxwell forensic DNA isolation kit (Promega, Leiden, The Netherlands) according to manufacturer’s protocol. Pre-amplification was accomplished for enrichment of the target DNA^15^. Genotypes of *PvuII* in *ESR1* (rs2234693) were established using commercially available pre-designed Taqman assays obtained from Applied Biosystems (Nieuwerkerk aan den IJssel, the Netherlands). Endpoint genotyping was performed on the LightCycler 480 Real Time PCR System (Roche, Almere, The Netherlands) according to standard procedures.

### Statistical analysis

A SNP call rate > 85% was found acceptable, as FFPE samples were used and therefore a low call rate could be expected. Genotype distributions were tested for the Hardy–Weinberg equilibrium (HWE) and chi-squared test were performed. Cox regression models were used to evaluate the effect of the SNP on covariates and DFS and OS. Hazard ratios (HRs) and 95% confidence intervals (95% CIs) were given. If the SNP was found to be (borderline) significant with a *p*-value of less than *0.1* in univariate Cox regression models it carried forward to a multivariate model. Risk factors found to have a *p*-value of less than *0.1* in univariate analyses were carried forward in the multivariate model as well. The median follow-up time was calculated by the reverse Kaplan–Meier methodology^16^. All tests were two tailed and *p*-values of less than *0.05* were considered significant. The data was analyzed using Statistical Package for Social Sciences (SPSS) software 23.0 (IBM Corp., Armonk, NY, USA).

### Ethical approval

All procedures performed in studies involving human participants were in accordance with the ethical standards of the central medical ethics review board of the Erasmus University Medical Center in Rotterdam, The Netherlands and with the 1964 Helsinki declaration and its later amendments or comparable ethical standards. This article does not contain any studies with animals performed by any of the authors.

### Informed consent

Informed consent was obtained from all individual participants included in the study.

## Results

### Patient characteristics

**Figure 1:**
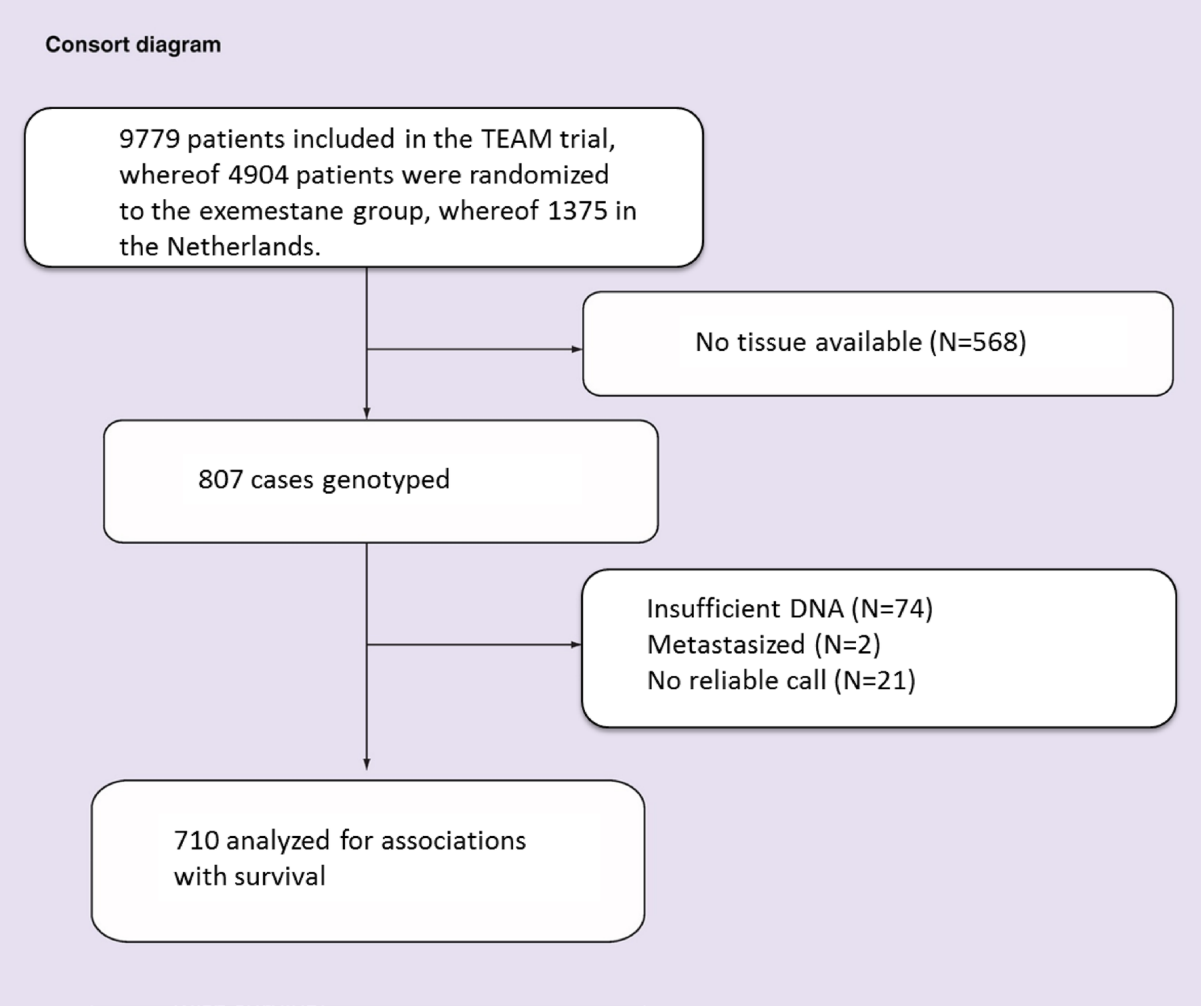
Consort diagram of the Dutch cohort of the TEAM trial: patients randomized to exemestane only.

From the 9779 patients enrolled in the international TEAM study, 2753 patients were included in the Netherlands, whereof 1374 were randomized to exemestane alone. FFPE tissue was available of 807 (58.7%) of the Dutch exemestane patients. Two patients were ineligible because of metastatic disease before they started treatment and were therefore excluded from analyses as summarized in the consort diagram (Fig. 1). No genotypes could be called due to insufficient DNA quality for 74 patients.

### Genotyping

The genotyping call rate for the rs2234693 SNP was 97%, as shown in Table 1. The genotyped samples (N = 731) were not significantly different from the whole group (N = 805) concerning age, BMI, TNM stage, tumor size, nodal status, histological grade of the tumor, progesterone status, type of operation (wide excision or mastectomy) and received adjuvant therapy, as determined by visually comparing continuous distributions and comparing frequencies for categorical variables, making informative drop-out unlikely (data not shown). Genotype frequencies were similar to those observed in a publicly available dataset from European subjects, with a Minor Allele Frequency (MAF) of the C allele of 0.464.

**Table 1:** Allelic frequencies of genotyped SNP.

rs-number	SNP	Allele	*n* (%)	HWE χ2	P value	Call rate (%)
*rs2234693*	ESR1, *PvuII*	TT	200 (28.2)	0.35	0.55	97%
		CT	361 (50.8)			
		CC	149 (21.0)			

ESR1, estrogen receptor-1; SNP, single nucleotide polymorphism.

### Survival analysis

**Figure 2:**
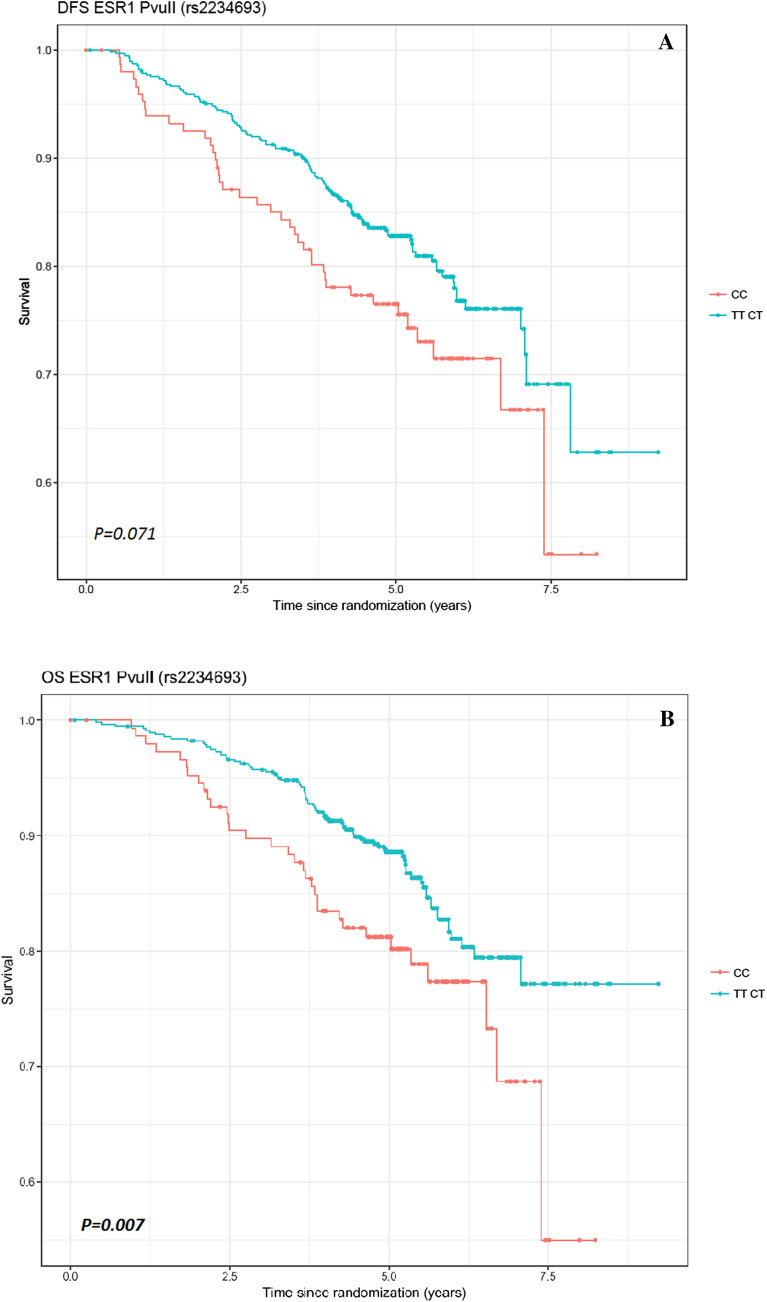
Kaplan–Meier curves of disease-free survival (**A**) and overall survival (**B**) for rs223469. *Note*: P-values are given for the multivariate analyses of the Cox regression analyses. DFS, disease free survival; OS, overall survival, ESR1, estrogen receptor 1.

The median follow-up for the genotyped samples of Dutch Exemestane patients was 5.22 years (95% CI 5.10–5.35). Kaplan–Meier curves are shown in Fig. 2. The estimated HRs and associated 95% CIs for univariate and multivariate Cox model analyses for DFS and OS, respectively, are shown in Tables 2 and 3. The risk factors T stage, PR status, histological grade and type of operation were associated with DFS, while T stage and type of surgery were associated with OS. The multivariate Cox models were adjusted for these covariates. The variant T allele of *PvuII* (rs2234693) in *ESR1* was associated with a better DFS (HR 0.689, 95% CI 0.480–0.989, *P* = 0.044) in univariate analysis, but not anymore in multivariate analysis (HR 0.696, 95% CI 0.469–1.031, *P* = 0.071). Moreover, the variant T allele of this SNP was associated with improved OS (HR 0.616, 95% CI 0.411–0.923, *P* = 0.019) in both univariate and multivariate analyses (HR 0.571, 95% CI 0.380–0.856, *P* = 0.007).

**Table 2:** Univariate and multivariate Cox models of DFS for different variables and rs2234693.

DFS	Frequency	Univariate			Multivariate		
		HR	95% CI	*P value*	HR	95% CI	*P value*
**Age**	805	0.997	0.980–1.015	*0.763*			
**BMI**	805	0.984	0.951–1.019	*0.360*			
**T stage**							
T1	367 (45.7)						
T2-4	436 (54.3)	2.002	1.434–2.796	***0.00005***	1.833	1.258–2.671	***0.002***
**N stage**							
N0	237 (29.5)						
N +	566 (70.5)	1.198	0.842–1.704	*0.315*			
**PR status**							
Positive	590 (77.1)						
Negative	175 (22.9)	1.684	1.198–2.367	***0.003***	1.716	1.120–2.434	***0.002***
**Histological grade (BR)**							
1	143 (18.7)	1.000		0.094*	1.000		0.150*
2	369 (48.4)	1.712	1.038–2.824		1.673	0.989–2.831	
3	251 (32.9)	1.691	1.002–2.854		1.621	0.931–2.821	
**Most extensive surgery**							
Wide local excision	372 (46.2)						
Mastectomy	433 (53.8)	1.742	1.258–2.413	***0.001***	1.527	1.057–2.206	***0.024***
**Adjuvant chemotherapy**							
No	589 (74.3)						
Yes	216 (26.8)	0.969	0.677–1.387	*0.865*			
**Adjuvant radiotherapy**							
No	487 (60.5)						
Yes	318 (39.5)	0.846	0.619–1.156	*0.294*			
**rs2234693 (ESR1, PvuII)**							
CC	149 (18.4)	1.000		0.105*			
CT	361 (51.1)	0.656	0.445–0.969				
TT	200 (28.1)	0.751	0.487–1.158				
*CC*	149 (21.0)	1.000			1.000		
*TT/CT*	561 (79.0)	0.689	0.480–0.989	***0.044***	0.696	0.469–1.031	***0.071***

DFS, disease free survival; HR, hazard ratio; CI, confidence interval; BMI, body mass index; PR, progesterone receptor; BR, Bloom Richardson. The multivariate analysis is compensated for T stage, PR status, histological grade, and type of surgery. Bold values indicate P < 0.05. *Global P-value.

**Table 3:** Univariate and multivariate Cox models of OS for different variables and rs2234693.

OS	Frequency		Univariate			Multivariate		
			HR	95% CI	*P value*	HR	95% CI	*P value*
**Age**	805		0.993	0.973–1.012	*0.457*			
**BMI**	805		0.969	0.929–1.010	*0.137*			
**T stage**								
T1	367 (45.7)							
T2-4	436 (54.3)		2.091	1.422–3.074	***0.0002***	1.803	1.200–2.708	***0.005***
**N stage**								
N0	237 (29.5)							
N +	566 (70.5)		1.270	0.845–1.910	*0.251*			
**PR status**								
Positive	590 (77.1)							
Negative	175 (22.9)		1.371	0.914–2.056	*0.127*			
**Histological grade (BR)**								
1	143 (18.7)		1.000		0.483*			
2	369 (48.4)		1.177	0.690–2.007				
3	251 (32.9)		1.383	0.797–2.398				
**Most extensive surgery**								
Wide local excision	372 (46.2)							
Mastectomy	433 (53.8)		1.875	1.285–2.734	***0.001***	1.548	1.040–2.306	***0.031***
**Adjuvant chemotherapy**								
No	589 (74.3)							
Yes	216 (26.8)		0.834	0.543–1.279	*0.405*			
**Adjuvant radiotherapy**								
No Yes	487 (60.5) 318 (39.5)		0.870	0.609–1.245	*0.447*			
**rs2234693 (ESR1, PvuII)**								
CC	149 (18.4)		1.000		0.027*	1.000		0.014 *
CT	361 (51.1)		0.546	0.350–0.852		0.515	0.330–0.805	
TT	200 (28.1)		0.748	0.463–1.208		0.672	0.415–1.087	
*CC*	149 (21.0)		1.000			1.000		
*TT/CT*	561 (79.0)		0.616	0.411–0.923	***0.019***	0.571	0.380–0.856	***0.007***

OS, overall survival; HR, hazard ratio; CI, confidence interval; BMI, body mass index; PR, progesterone receptor; BR, Bloom Richardson. The multivariate analyses are compensated for T stage and type of surgery. Bold values indicate P < 0.05. *Global P-value.

## Discussion

We found that the T genotype of *PvuII* (rs2234693) in *ESR1* was associated with a better OS and a trend for better DFS than the C genotype in postmenopausal, hormone receptor positive early breast cancer patients treated with adjuvant exemestane alone in the TEAM study. We previously described the association of the TT and TC genotypes (versus CC genotype) of *PvuII* in *ESR1* and a better survival in a comparable subgroup of Dutch TEAM patients treated with tamoxifen^9^. Therefore, our results are more consistent with a prognostic factor, rather than a predictive drug response effect, as this SNP is related to OS in early breast cancer patients for both types of endocrine treatment (exemestane or tamoxifen). Particularly, because exemestane and tamoxifen differ in working mechanism^17^.

Our results are in line with those of Gabrinski et al. who found an association between the *ESR1 PvuII* genotype and DFS regardless of type of endocrine treatment, however final data are not yet published^18^.

The *PvuII* restriction site is localized in intron 1 of the *ESR1*, 400 basepairs upstream of exon 2^13^. Loss of the *PvuII* restriction site due to T- > C transition, may result in a binding site for transcription factor B-Myb, which amplifies transcription of the estrogen receptor^19,20^. This upregulation of the estrogen receptor possibly explains the association of the C-allele with worse survival. Alternatively, this SNP may be in linkage disequilibrium with causal synonymous polymorphisms elsewhere in the *ESR1* or another gene^19^.

A limitation of our study is that DNA from FFPE samples is not ideal for genotyping and samples with intact genomic DNA as blood or frozen tissue would be preferred, however, this was not available in our study, and earlier research showed that FFPE tumor tissue and normal tissue was highly concordant^21,22^. Although there are discordant results described in the literature, for example the deletion of the *CYP2D6* gene in breast tumor tissue is reported to cause departures from HWE^23^, it seems unlikely that the *ESR1* gene is deleted in our study as HWE is not violated (Table 1).

As the C allele of rs2234693 is a frequently observed allele with a MAF of 0.464 in our study, this SNP is of interest and may be validated in other large studies. Moreover, to personalize endocrine therapy in breast cancer in the future a 18F‐fluoroestradiol (FES) positron emission tomography (PET) may be used for whole‐body imaging for receptor status assessment to research the phenotype-genotype of the *PvuII* in *ESR1*^24^.

## Conclusions

The T allele of *PvuII* (rs2234693) in the *ESR1*gene is associated with improved overall survival in postmenopausal, hormone receptor positive early breast cancer patients and may be considered as a prognostic marker in early breast cancer. Further studies into the prognostic value of this biomarker are warranted.

## Abbreviations

DFS: Disease-free survival
ESR1: Estrogen receptor-1
FES: 18F‐fluoroestradiol
FFPE: Formalin-fixed paraffin embedded
HR: Hazard ratio
HWE: Hardy–Weinberg equilibrium
MAF: Minor Allele Frequency
OS: Overall survival
PET: Positron emission tomography
SNP: Single nucleotide polymorphism
SPSS: Statistical package for social sciences
TEAM: Tamoxifen exemestane adjuvant multinational
